# Recent Progress in Hydrogel-Based Synthetic Cartilage: Focus on Lubrication and Load-Bearing Capacities

**DOI:** 10.3390/gels9020144

**Published:** 2023-02-08

**Authors:** Fei Qiu, Xiaopeng Fan, Wen Chen, Chunming Xu, Yumei Li, Renjian Xie

**Affiliations:** 1 Key Laboratory of Prevention and Treatment of Cardiovascular and Cerebrovascular Diseases, Ministry of Education, Gannan Medical University, Ganzhou 341000, China; 2 School of Rehabilitation Medicine, Gannan Medical University, Ganzhou 341000, China; 3 Key Laboratory of Biomaterials and Bio-Fabrication in Tissue Engineering of Jiangxi Province, Ganzhou 341000, China; 4 School of Materials Science and Engineering, South China University of Technology, Guangzhou 510641, China; 5 National Engineering Research Center for Tissue Restoration and Reconstruction, South China University of Technology, Guangzhou 510006, China; 6 School of Basic Medicine, Gannan Medical University, Ganzhou 341000, China; 7 School of Medical Information Engineering, Gannan Medical University, Ganzhou 341000, China

**Keywords:** articular cartilage, hydrogels, implants, lubrication, load-bearing

## Abstract

Articular cartilage (AC), which covers the ends of bones in joints, particularly the knee joints, provides a robust interface to maintain frictionless movement during daily life due to its remarkable lubricating and load-bearing capacities. However, osteoarthritis (OA), characterized by the progressive degradation of AC, compromises the properties of AC and thus leads to frayed and rough interfaces between the bones, which subsequently accelerates the progression of OA. Hydrogels, composed of highly hydrated and interconnected polymer chains, are potential candidates for AC replacement due to their physical and chemical properties being similar to those of AC. In this review, we summarize the recent progress of hydrogel-based synthetic cartilage, or cartilage-like hydrogels, with a particular focus on their lubrication and load-bearing properties. The different formulations, current limitations, and challenges of such hydrogels are also discussed. Moreover, we discuss the future directions of hydrogel-based synthetic cartilage to repair and even regenerate the damaged AC.

## 1. Introduction

Articular cartilage (AC) is avascular and aneural connective tissue that covers the ends of diarthrodial joints to maintain frictionless movement without pain [1]. Since the AC in the knee joint operates in the most mechanically stressed conditions and has attracted special attention during the last decades due to its remarkable mechanical properties [2,3], AC in the following section particularly refers to the knee cartilage. AC, which has four anatomically distinct zones, is mainly composed of chondrocytes (less than 10% of the volume) and the extracellular matrix (ECM) secreted by chondrocytes [4]. Apart from water (accounting for 65–80% of the total weight), the ECM primarily consists of collagen (predominately type II collagen), proteoglycans, glycoproteins, and non-collagenous proteins [5]. The unique structure and composition of AC endow it with its main functions or properties, that is, resisting compressive forces and providing the most lubricated surface known to humans when the compressed cartilages slide past each other during routine activities [1,6].

Osteoarthritis (OA), the most common joint disease, is characterized by the progressive degradation of AC [7]. Approximately 300 million people suffer from OA worldwide and it has been listed as the second-leading cause of disability by the World Health Organization [8]. Dysfunction of the above-mentioned two properties, especially the lubricating capacity, caused by accidental trauma or aging, leads to the onset of OA [9]. Thus, emerging cartilage-inspired biolubricants that can resurface the OA-damaged cartilage to decrease the friction of AC and subsequently alleviate the conditions of OA have been developed [10,11,12]. We have summarized the recent progress of this lubrication-based strategy previously [13]. However, lubrication-based therapies are not available when the AC is degraded significantly or large AC defects are observed. As we know, OA can be classified into pre-OA, early-OA, progressive-OA, and end-stage OA based on the degradation degree of AC, and, therefore, partial AC defects and osteochondral defects can be observed accordingly [14]. Unfortunately, pre-OA is clinically undetectable and the degradation of AC is irreversible once OA initiates. Cartilage repair and even total knee arthroplasty, which requires lubrication and load-bearing capacities simultaneously, may still be the best option for the treatment of OA [15].

Hydrogels, which are prepared by physically or chemically crosslinking hydrophilic polymers or nonpolymeric networks, have been widely used for partial cartilage repair with or without embedded cells due to their structural and functional similarity to AC [16,17,18,19]. Currently, hydrogels are usually adopted for cartilage repair in several typical approaches: (1) chondrocytes or stem cells are encapsulated within hydrogels and then transferred to the defect site, where hydrogels are used as scaffolds [20,21]; (2) hydrogels, usually fabricated as nanogels or microgels, are used as carriers of some specific drugs and/or growth factors that can promote the regeneration of cartilage so that these drugs or growth factors can be released in a controlled manner [22,23]; (3) hydrogels are used as cartilage substitutes or synthetic cartilage that can be implanted into the targeted area by injection or open surgery, where the hydrogels with high strength and low friction function as “cartilage” directly [24,25,26]. The progress of the first two approaches has been reviewed systemically elsewhere [27,28,29], whereas the third approach has also made important developments, but few summary reports have been published. Therefore, in this review, hydrogel-based synthetic cartilage, lubrication, and high strength are adopted as keywords to screen the research published during the last five years. Starting with an overview of the structure and properties of AC, the lubrication, and load-bearing properties as the two key features of synthetic cartilage are discussed, followed by the treatment options for AC defects in order to better understand the application of hydrogels used as synthetic cartilage. Then, we review the typical progress in this use of hydrogels inspired by AC components and/or structure and finally discuss the future directions.

## 2. Articular Cartilage (AC)

Articular cartilage is a complex bio-hydrogel with a two-phase structure comprising liquid and solid phases. The liquid phase plays a role in reducing interfacial friction and bearing most of the load, as well as the transport of nutrients, while the solid phase can achieve wear resistance under high load conditions [4]. The biological and biomechanical properties of AC rely heavily on the integrity of AC.

### 2.1. Structure and Properties of AC

**Figure 1:**
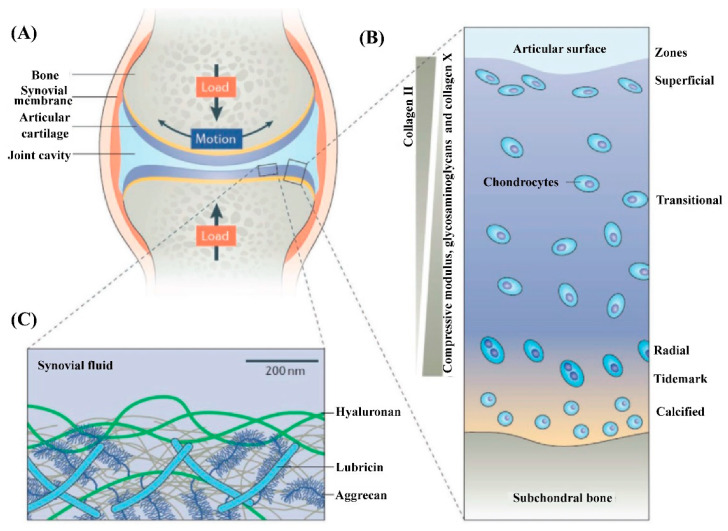
Illustration of the components and structure of AC. (**A**) The knee synovial joint is mainly composed of the synovial membrane, AC, and the synovial fluid within the synovial cavity. (**B**) AC is characterized by its layered structure. Chondrocytes make up less than 5% (volume fraction) of AC. The main composition of ECM, type II collagen, glycosaminoglycans, collagen X, and the depth-dependent modulus, are indicated. (**C**) Illustration of the outer surface of AC that determines the lubrication performance of AC. Glycosaminoglycans, including hyaluronic acid (HA) and aggrecan, as well as lubricin and phospholipids (are not shown here) synergically assemble to form a lubrication layer outer of the AC surface to determine its remarkable lubrication at high pressure. Reprinted with permission from Ref [4]. Copyright 2021, Wiley-VCH.

Microscopically, as shown in Figure 1, three distinct zones can be distinguished from the surface of AC to the bottom based on the orientation of type II collagen fibers, which are the structural and fibrillar protein that provides AC with its strength [13,30,31,32]. Among these three zones, the superficial zone accounts for approximately 10–20% of the volume of AC and is the thinnest layer, where the type II collagen fibers align parallel to the outer surface of the AC. Therefore, the superficial zone can best resist subjected stresses (compress, tensile, and shear). Within the superficial zone, there are some important molecules, that is, lubricin, aggrecan, and hyaluronic acid (HA), combined with phospholipids (having a specific high affinity for HA), that maintain the remarkable lubrication of AC due to the boundary lubrication mechanism [33,34,35]. Many researchers have noted that the disruption of the superficial zone leads to biomechanical (e.g., increase in cartilage friction) and biological (e.g., increased secretion of interleukin 1β) changes in AC, thus determining the onset of OA [36,37,38]. The middle or transitional zone, representing 40–60% of AC volume, consists of collagen fibers with larger diameters and oblique organizations, as well as a higher concentration of proteoglycans and chondrocytes when compared with those within the superficial zone [39,40]. Generally, the biomechanical function of the middle zone is to transit the compressive and shear stresses to the deep zone, where the collagen fibers with the largest diameter align perpendicular to the surface of AC. The deep zone, accounting for the other approximately 30% of the AC, has the function of facilitating the load distribution and empowering most of the compressive stresses [41]. Within these zones, there are sparsely distributed chondrocytes (the only type of cell in AC). Although the quantity of chondrocytes only occupies at most 5% of the volume of AC, the integrity of AC relies heavily on the metabolism of chondrocytes to degrade the aged or damaged ECM and secrete new ECM to maintain the structural and functional integrity of AC [37,42]. The phenotype of chondrocytes varies from the superficial zone to the deep zone. The superficial zone usually contains flattened ellipsoid chondrocytes, while the chondrocytes in the middle zone are spherical and those in the deep zone are spheroid-shaped [43]. Generally, the different phenotypes of chondrocytes reflect the different metabolic level, which decreases from the superficial zone to the deep zone. Proteoglycans, another dominant component of AC, are distributed within the network formed by collagen fibers and are conducive to retaining water within the ECM due to the abundant highly hydrophilic nature of proteoglycans. From the surface to the deep zone of AC, the content of proteoglycans tends to increase and the water content tends to decrease (most of the water inside AC is free water not bound water). Underneath the deep zone is the tidemark and calcified zone (also thought to be the fourth zone of AC), which serve as a barrier to prevent the invasion of blood vessels into the AC from subchondral bone [44].

The special structure and composition of AC determine its complicated biological and biomechanical properties. The paralleled type II collagen within the superficial zone protects the chondrocytes underneath from the shear stress, and the vertical type II collagen within the deep zone, combined with the proteoglycans interpenetrated within the collagen network, endows the AC with its load-bearing properties. Some key mechanical parameters of AC are its modulus (0.1–2.0 MPa, increases with the depth), its stiffness (≥1 MPa, increases with the depth), its compressive capacity (14–59 MPa, increases with the depth), and its Poisson’s ratio (0.06–0.30) [45,46].

### 2.2. AC Defects and the Strategies for AC Repair

**Figure 2:**
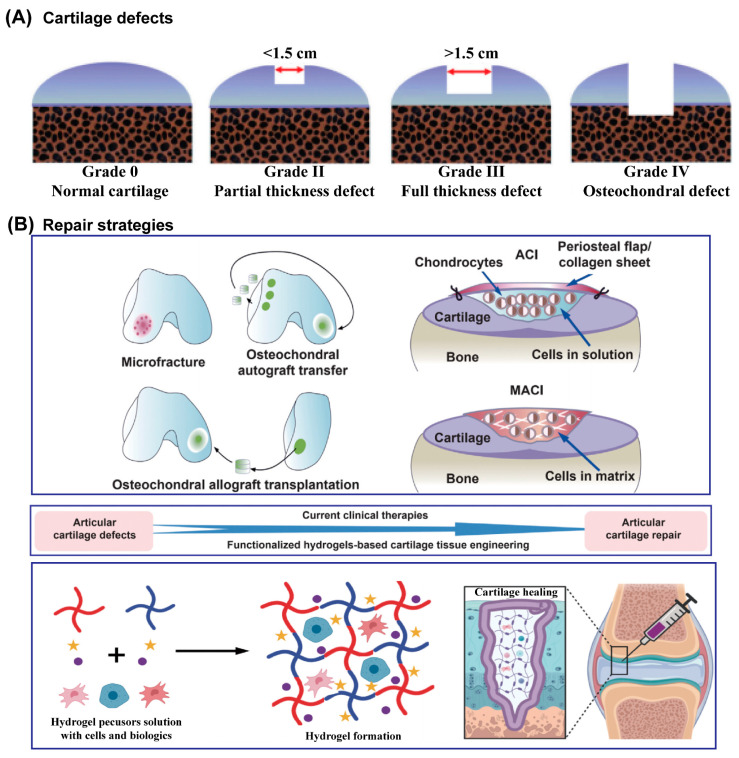
Schematic illustration of ICRS classification system of AC defects (**A**) and schematic diagram of traditionally used clinical repair strategies and hydrogel-based AC tissue engineering for AC defects (**B**). Reprinted with permission from Ref [28]. Copyright 2022, Elsevier.

AC damage usually results from trauma due to AC overload, which then increases the friction of AC, and the initial series of biological activities aggravate and perpetuate the AC defects [47,48]. Due to its very limited self-healing ability, once AC has incurred damage, the development of AC defects is irreversible until the AC is completely disrupted, and then the damage involves the underlying subchondral bone [1], as shown in Figure 2A. Currently, there are two major categories of AC defects: partial defects and full-thickness AC defects [1,14,15]. Generally, partial defects refer to damage to the above-mentioned three zones of AC, and full-thickness defects refer to damage or injury that penetrates these zones and the subchondral bone to reach the cancellous bone. It is worth noting that there are nerves and blood vessels inside cancellous bone, so much more attention should be paid when repairing cartilage at this stage, such as the spontaneous immune response [49]. Many comprehensive grading systems have been formulated to quantitatively evaluate cartilage injury. Among them, commonly used scoring systems have been established by the Osteoarthritis Research Society International (OARSI) and the International Cartilage Repair Society (ICRS) [50,51,52]. The scores have demonstrated high reliability and internal correlation.

Numerous techniques have been developed to repair AC defects (Figure 2B), especially partial defects. However, none of them, individually or in combination, can provide satisfactory or reliable, and most importantly long-term efficacy, due to the complicated and special operational environment of AC [53]. Clinically, microfracture [54], osteochondral autograft and allograft, autologous chondrocyte implantation (ACI), and matrix-assisted autologous chondrocyte implantation (MACI) are the main approaches for AC defects [55,56,57]. However, the existence of fibrocartilage, not AC, triggered the further optimization of treatments and the development of new strategies. Hydrogels, containing high water content and devisable or tunable mechanical properties, have widely been used as scaffolds or carriers in tissue engineering approaches to repair cartilage. Most hydrogels are designed as injectable hydrogels so that they can fill the irregular defect site appropriately. Though this approach has been of great interest to researchers, hydrogels with adequate biological and physicochemical capacities are yet to succeed.

Recently, synthetic or artificial cartilage derived from biomimetic hydrogels with robust load-bearing and/or lubrication properties has made significant progress due to its advantages (such as evading the fibrocartilage with undesirable biomechanics) compared with hydrogels encapsulating cells. Thus, it might enrich our understanding and lead to a new method of AC repair.

## 3. Cartilage-Inspired Hydrogels for AC Repair

The structure and composition of AC endow it with excellent mechanical properties, so the mechanical behaviors of hydrogels, especially their lubrication and load-bearing capacities, play a crucial role in determining the quality of the repair. Hydrogels inspired by AC focus on structure (“layer” or zonal structure) and/or composition (components of ECM or AC) mimicking to achieve mechanical mimicry. For these hydrogels to be used as cartilage implants or grafts, the cartilage-like features, load-bearing functions with compressive strengths from 14 MPa to 59 MPa and extremely low friction (with friction coefficients as low as 0.001), are of paramount importance. Very recently, various hydrogels aiming at improving these two properties, individually or in combination, have been designed and prepared. In this section, we focus on discussing the recent progress of such hydrogels inspired by the components or structure of AC.

### 3.1. Cartilage-Component-Inspired Hydrogels

AC principally consists of water, collagen type II fibers, and negatively charged proteoglycans, including hyaluronic acid and aggrecan. The network formed by type II collagen fibers gives AC high tensile strength, and the aggrecan retains large amounts of water within the AC due to the highly hydrophilic sulfate groups [58]. Additionally, aggrecan molecules are tethered to hyaluronic acid to form brush-like aggregates [59] which are trapped within collagen networks, leading to the osmotic pressure that enables AC to resist compressive loads and increase its load-bearing capacity. Inspired by these components and their roles in AC, alone or in combination, cartilage-like hydrogels have recently been designed.

#### 3.1.1. Proteoglycan-Inspired Hydrogels

A.K. Means et al. prepared a double-network hydrogel with poly(2-acrylamido-2-methylpropanesulfonic acid) (PAMPS) as the first network and poly(N-isopropylacrylamide-co-acrylamide) as the second network. Due to the highly hydrophilic nature and double-network structure of poly(AMPS) [60], this hydrogel not only simultaneously achieved high compressive strength (~23 MPa, similar to cartilage), cartilage-like modulus (~1 MPa), and water content (~80%) but also showed a 50% reduction in the friction coefficient when compared with healthy porcine cartilage. Similarly, P.E. Milner et al. designed a triple network hydrogel [61] in which PAMPS was polymerized as the first network and then copolymerized with acrylamide (AAm) to prepare the poly(AMPS)-co-poly(AAm) double-network hydrogel. Subsequently, poly(2-methacryloyloxyethyl phosphorylcholine) (PMPC) was further incorporated to form a triple-network hydrogel. The presence of the third network of PMPC (serving as a biomimetic boundary lubricant), combined with an ultra-tough double network, replicated both the boundary and biphasic lubrication of AC, and thus had a yield stress of 26 MPa (an order of magnitude higher than that found in human knee AC) and significantly further decreased the friction coefficients due to the superhydrophilicity of MPC.

**Figure 3:**
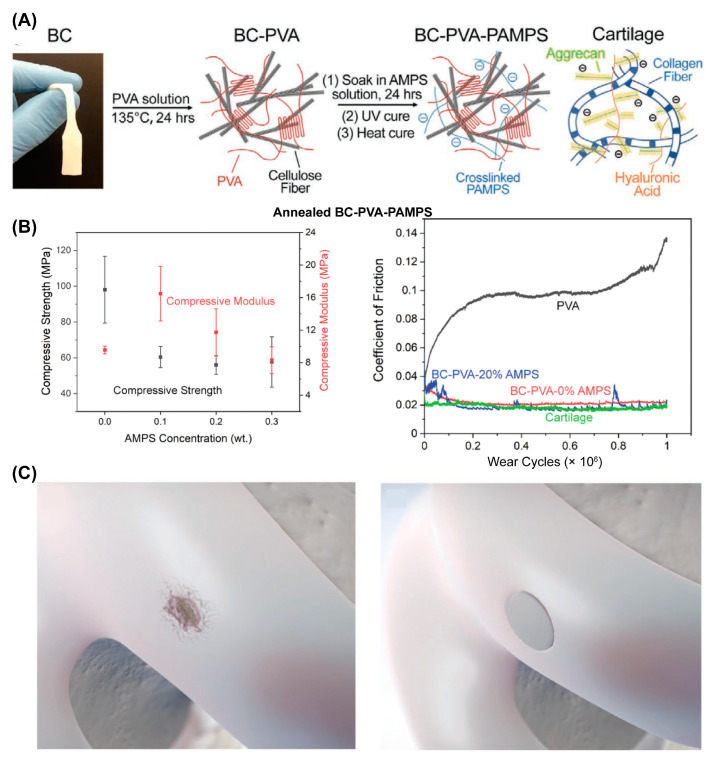
Synthetic hydrogel composites with mechanical strength comparable to or even greater than AC. (**A**) Illustration of the BC–PVA–PAMPS hydrogel fabrication process. Reprinted with permission from Ref [62]. Copyright 2020, Wiley-VCH. (**B**) Compressive strength and compressive modulus of annealed BC–PVA–PAMPS hydrogel (mean ± SD), and the friction coefficient of annealed BC–PVA–PAMPS hydrogels sliding against AC. (**C**) Illustration of treatment of AC defect using annealed BC–PVA–PAMPS hydrogel. Panels (**B**,**C**) are reprinted with permission from Ref [63]. Copyright 2022, Wiley-VCH.

F. Yang et al. reported another cartilage-inspired hydrogel that involved introducing bacterial cellulose (BC) into a poly(vinyl alcohol) (PVA)-PAMPS double-network hydrogel [62] (referred to as the BC–PVA–PAMPS hydrogel). As shown in Figure 3A, the BC offered tensile strength in a manner just like the collagen network in AC, while PAMPS played a similar role to aggrecan in AC. The BC–PVA–PAMPS exhibited a cartilage-like compressive modulus (23 MPa) and strength (10.8 MPa) and cartilage-matching time-dependent deformation. Importantly, the friction coefficient of BC–PVA–PAMPS hydrogel against a stainless-steel pin was 0.06 under the pressure of 1 MPa measured with the pin-on-disk method, which was significantly lower than that of porcine cartilage against a stainless-steel pin (0.11) measured using the same procedure. Very recently, they further improved the strength of BC–PVA–PAMPS by changing the freeze–thaw to the annealing process to increase the crystallinity of PVA [63]. The obtained annealed BC–PVA–PAMPS hydrogel showed a tensile strength of 51 MPa and a compressive strength of 98 MPa (Figure 3B, left), which were greater than those of AC (40 and 59 MPa). The friction coefficient of annealed BC–PVA–PAMPS against AC was similar to that of AC against AC under the pressure of 1 MPa as measured with the pin-on-disk method (Figure 3B, right). Moreover, they demonstrated the ability of such a hydrogel attached to a titanium implant (with a shear strength greater than that of AC on bone) to treat AC defects (Figure 3C). 

#### 3.1.2. Phospholipid-Inspired Hydrogels

**Figure 4:**
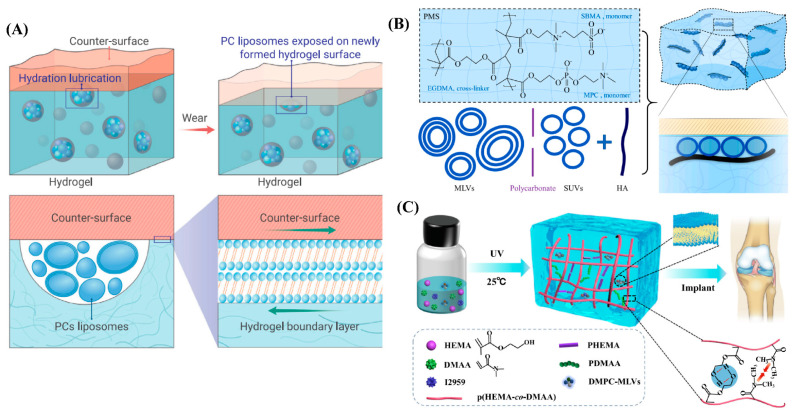
Phospholipid-inspired cartilage-like hydrogels. (**A**) Illustration of the self-lubricating and lipid-incorporated hydrogel. The incorporated lipids formed micro-reservoirs throughout the gel bulk, and additional micro-reservoirs were exposed due to friction, which enabled the boundary lubrication layer of the lipids to form on the surface, leading to a reduction in friction via hydration lubrication. Reprinted with permission from Ref [67]. Copyright 2022, Elsevier. (**B**) Schematic illustrating of the formation PMS-HSPC(SUV)-HA hydrogel and the synergistic lubrication mechanism. The super-lubricated state after the incorporation of lipid SUV and HA was mainly attributed to the synergistic lubrication effect between lipids and HA after the formation of uniformly arranged lipid liposomes around the HA structure. Reprinted with permission from Ref [65]. Copyright 2022, Elsevier. (**C**) Schematic illustration of the synthesis of lipid-lubricated hydrogels with biocompatible, high-strength lipid-lubrication performance. Reprinted with permission from Ref [66]. Copyright 2022, Elsevier.

Recently, Lin et al. prepared self-lubricating hydrogels by incorporating dimyristoylphosphatidylcholine (DMPC) and hydrogenated soy phosphatidylcholine (HSPC) in the form of multilamellar vesicles (MLVs) into the poly(hydroxyethylmethacrylate) (PHEMA) hydrogel [64], as illustrated in Figure 4A. A back-and-forth mode was used to estimate the friction between the hydrogel and a polished stainless-steel surface at 25 °C (room temperature) and 37 °C (physiological temperature) over different loads or contact pressures. The results showed that an 80 to 99.3% reduction in friction and wear could be observed when compared with the lipid-free PHEMA hydrogel. The friction coefficients arising from lipid-incorporating hydrogels ranged from ~0.02 at lower pressures to 0.005 at higher pressures, in contrast, much higher friction was observed (0.5 ≲ μ ≲ 1) for lipid-free hydrogels, especially when the pressure was higher than 0.5 MPa. Furthermore, they found that extremely low friction at a high pressure (1.10 MPa) could be maintained when the lipid-incorporating hydrogels were fully dried and then hydrated. Due to such highly effective lubrication, the wear of lipid-containing PHEMA hydrogel was only 9 ± 3 μm after sliding for 1 h at a pressure of 1.53 MPa with μ ≈ 0.01, which remained unchanged throughout. Lipid-free PHEMA gel, meanwhile, was destroyed after sliding for only a few seconds in the same conditions. Interestingly, they demonstrated that reduction in friction and wear was attributed to the continuous self-renewal of the molecularly thin, lipid-based boundary layer between the sliding surfaces (hydrogel and polished stainless surfaces) as friction abraded the hydrogel, and thus progressively exposed the incorporated lipids during sliding. This self-lubricating hydrogel shows its great potential to apply as artificial cartilage if it can be designed as a personalized shape to match the irregular AC defects. Similarly, Feng et al. prepared another super-lubricated hydrogel by incorporating HSPC in the form of MLVs or single unilamellar vesicles (SUVs) and hyaluronic acid (HA) within the poly(2-methacryloyloxyethyl phosphorylcholine) (MPC)-co-poly(sulfobetaine methacrylate) (SBMA) hydrogel (PMS hydrogel). Thus, PMS-HSPC(SUV)-HA and PMS-HSPC(MLV)-HA hydrogels were obtained [65]. As shown in Figure 4B, the friction coefficient of PMS-HSPC(SUV)-HA significantly reduces to 0.0052 ± 0.0019 when sliding against a Si_3_N_4_ ball under the load of 1N, and the compressive strength of PMS-HSPC(SUV)-HA was also significantly enhanced to 0.072 MPa when compared with those of the PMS hydrogel. However, the pressure applied for friction coefficient measurement and the compressive strength are still insufficient for a cartilage implant. Very recently, Xiao et al. also designed a lipid-lubricated hydrogel by incorporating DMPC-MLVs within the copolymer consisting of HEMA and N, N-dimethylacrylamide (DMAA) (p(HEMA-co-DMAA)) [66], as shown in Figure 4C. The lipid-containing p(HEMA-co-DMAA) hydrogel showed outstanding properties in stiffness and load-bearing, and the maximum compressive strength and compressive modulus could be reached at 5.8 MPa and 4.7 MPa, respectively, when the molar concentration ratio of HEMA to DMAA was 3:1. Meanwhile, the friction coefficient of the hydrogel could be as low as 0.026 under a load of 5N when sliding back and forth against stainless-steel ball. However, when the applied load was increased to 30 N, the friction coefficient increased to ~0.2, which perhaps limits its application as a cartilage substitute considering the high pressure in the knee joint.

### 3.2. Cartilage-Structure-Inspired Hydrogels

AC is typically characterized by its layer structure and its extraordinary tribological performance arising from the outer surface of the AC, where the synovial fluid stored within it can be extruded to form a lubricating layer composed of hyaluronic acid, phospholipids, and lubricin to reduce the friction when the AC is pressured and articulated. Additionally, underneath the lubrication layer, the collagen fibers within the middle zone and deep zone provide high load-bearing capacity. From the perspective of bionic design, layer hydrogels with excellent lubrication and load-bearing properties have recently been fabricated as artificial cartilage.

**Figure 5:**
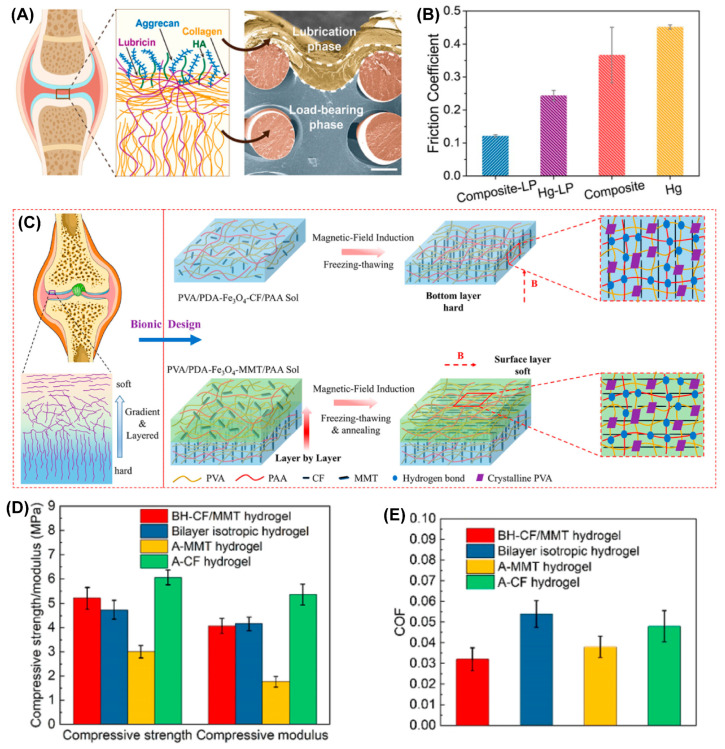
Typical cartilage structure-inspired hydrogels. (**A**) The main components consisted of an AC lubrication system within the AC superficial layer (left). The SEM cross-sectional morphology of Composite-LP, which clearly shows the load-bearing phase and lubrication phase (right). (**B**) The friction coefficients of the Composite-LP, Hg-LP, Composite, and Hg samples (load, 1 N; frequency, 1 Hz). Panels (**A**,**B**) are reprinted with permission from Ref [25]. Copyright 2022, American Chemical Society. (**C**) Schematic illustration of the bilayer-oriented heterogeneous hydrogel (BH-CF/MMT hydrogel). (**D**) The compressive strength and compressive modulus (**D**) and the average friction coefficient (**E**) of the bilayer-oriented heterogeneous hydrogel compared with control groups (bilayer unoriented hydrogel, A-MMT hydrogel, and A-MMT hydrogel). Reprinted with permission from Ref [70]. Copyright 2022, American Chemical Society.

Zhou’s group developed a series of robust, stiff, and wear-resistant hydrogels by grafting hydrophilic polyelectrolyte onto the subsurface of a stiff hydrogel to prepare the bilayer structure. These hydrogels showed cartilage-like features with high strength and low friction [24,26,68,69]. Zhao et al. from Zhou’s group very recently designed a composite hydrogel composed of a load-bearing phase and a lubrication phase by chemically grafting a thick highly hydrophilic poly (3-sulfopropyl methacrylate potassium) layer (lubrication phase) onto the subsurface of three-dimensional (3D) elastomer scaffold-hydrogel matrix [25] (load-bearing phase). As shown in Figure 5A,B, the integration of the 3D elastomer network not only acted as structural support to disperse the applied stress through a non-dissipative mechanism but also stored much more elastic strain energy via elastic deformation, which further enhanced its load-bearing properties. The highly hydrated lubrication phase gave the composite hydrogel lower average friction coefficients at a given load (1 N) or dynamic loads (from 0.2 N to 4 N) under a wide range of shear frequencies when sliding against a polydimethylsiloxane sheet using the pin-on-disk contact mode. Thus, the robust bulk load-bearing combined with good surface lubrication provides us with a new candidate for cartilage repair. Very recently, Chen et al. proposed a new strategy for preparing a bilayer anisotropic hydrogel with a horizontal orientation within the upper layer and a vertical orientation within the bottom layer [70], which is similar to the orientation of collagen fibers in the superficial zone and deep zone of AC. This allowed for low friction and high load-bearing strength. As shown in Figure 5C–F, the magnetic polydopamine-Fe_3_O_4_-carbon fiber (PDA-Fe_3_O_4_-CF) nanohybrids were aligned inside the poly(vinyl alcohol) (PVA)/polyacrylic acid (PAA) hydrogel matrix by applying a magnetic field to prepare the bottom layer, then polydopamine-Fe_3_O_4_-montmorillonite (PDA-Fe_3_O_4_-MMT) nanohybrids were embedded within PVA/PAA hydrogel as the upper layer was constructed on the bottom layer to obtain the bilayer oriented heterogeneous hydrogel (BH-CF/MMT) after freeze-drying and annealing. When BH-CF/MMT slid against itself or against cartilage or cartilage slid against cartilage under a load of 30 N and sliding speed of 5 mm/s, four different contact friction pairs were compared, as shown in Figure 5E; the average friction coefficients were 0.028, 0.032, 0.043, and 0.046, respectively, which indicated cartilage-like lubrication performance of the BH-CF/MMT. Combined with the high compressive strength (5.21 ± 0.45 MPa) and compressive modulus (4.06 ± 0.31 MPa), BH-CF/MMT exhibited great prospects as a substitute for AC.

### 3.3. Cartilage Components and Structure-Inspired Hydrogels

**Figure 6:**
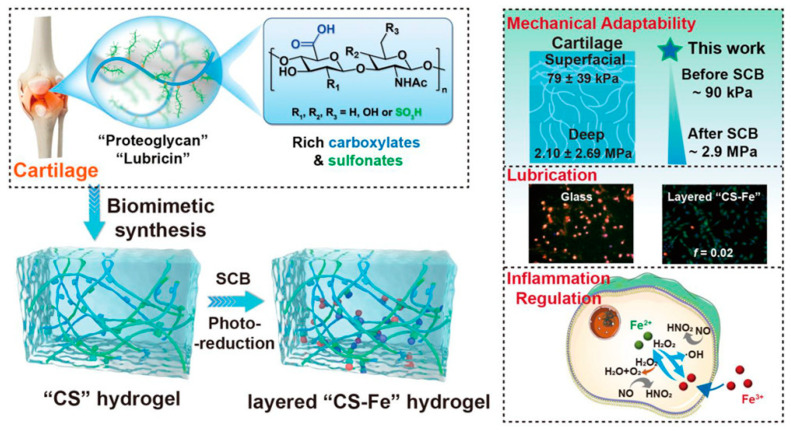
Preparation of cartilage components (proteoglycans and lubricin) and layer structure-inspired “CS” and layer “CS-Fe” hydrogels and their main functions, including mechanical adaptability, low friction, and inflammation regulation. Reprinted with permission from Ref [71]. Copyright 2022, American Chemical Society.

Inspired by AC rich in anionic proteoglycan contents and layer structure, Yu et al. fabricated a polyanionic hydrogel that contained rich carboxylates/sulfonates (CS) derived from acrylic acid (AAc, rich in carboxylate groups) and 3-sulfopropyl methacrylate potassium salt [71] (SPMK, rich in sulfonate groups), as shown in Figure 6. AAc and SPMK (the ratio was 4:1) were first polymerized directly using UV light, then the C_4_S_1_ hydrogel was immersed in 0.1 M ferric solution to achieve swelling crosslinking balance (SCB) due to the high affinity of ferric ions (Fe^3+^) for carboxylates. Thus, a competition for swelling (adsorption of water) and crosslinking (crosslinking between Fe^3+^ and carboxylate) existed when water molecules and Fe^3+^ penetrated the C_4_S_1_ hydrogels. Therefore, a mechanically strengthened C_4_S_1_-Fe hydrogel was obtained thanks to the second crosslinking of Fe^3+^. Importantly, this hydrogel showed adaptive mechanical properties due to the gradient concentrations of Fe^3+^ along the depth of the hydrogel from the surface, which further mimicked and matched the zone-dependent mechanical properties of AC. As shown in Figure 6, the Young’s moduli of AC in the superficial and deep zones were 79 ± 39 kPa and 2.10 ± 2.69 MPa, respectively, whereas the Young’s moduli of the C_4_S_1_ hydrogel before and after SCB were ~90 kPa and ~2.9 MPa, respectively. However, although the second crosslinking by Fe^3+^ enhanced the load-bearing performance, the hydration lubrication was compromised because the carboxylate and sulfonate groups were associated with Fe^3+^, and thus decreased the number of these two groups that were able to bond water to enhance hydration lubrication. Further inspired by the layer structure of AC, the authors exposed the C_4_S_1_-Fe hydrogel to UV irradiation to reduce the Fe^3+^ to Fe^2+^, then Fe^2+^ dissociated from the carboxylate and sulfonate due to its weaker affinity than Fe^3+^ for the CS groups, and thus a loose and highly hydrated top layer appeared without significant sacrifice of the compressive modulus. Accordingly, the friction coefficient decreased sharply from above 0.636 to 0.02 under a high load of 28 gf, and apoptosis of chondrocytes was avoided during sliding. They further found that the C_4_S_1_-Fe hydrogel could protect chondrocytes/fibroblasts from aggressive inflammation by suppressing the overexpression of hydroxyl radicals and nitric oxide. This hydrogel with adaptive mechanical and excellent lubrication properties inspired by the components and layer structure of AC showed great potential for cartilage repair, especially in the inflammatory OA environment.

Rong et al. designed a cartilage-mimicking hydrogel by grafting a thick hydrophilic layer onto the surface of a stiff hydrogel substrate [24]. Acrylic acid (AAc), acrylamide (AAm), and 2-(2-bromoisobutyryloxy) ethyl methacrylate (BrMA) were first polymerized via free radical polymerization to obtain a poly(AAm-AAc-BrMA) hydrogel, followed by second physical crosslinking in a Fe^3+^ solution to prepare the high-strength hydrogel (named as HHy-Br) substrate. Then, sulfate-rich monomers, (2-(methacryloyloxy) ethyl) dimethyl-(3-sulfopropyl) ammonium hydroxide (SBMA) or (3-sulfopropyl methacrylate potassium (SPMA) was allowed to polymerize in the subsurface to form a thick hydrophilic layer on the surface of HHy-Br, and thus prepared gradient and layered hydrogels: HHy-*g*-PSBMA and HHy-*g*-PSPMA. The top hydrophilic layer enables effective lubrication, whereas the bottom stiff layer offers load-bearing capacity. The friction coefficients of the obtained HHy-*g*-PSBMA and HHy-*g*-PSPMA hydrogels can reach the order of 0.01 under extremely harsh measurement conditions (contact pressure: 8.5 MPa). Particularly, the friction coefficient maintains its low level when the contact pressure increases to 10 MPa without obvious wear. Compared with other work, perhaps further biocompatibility characterization is needed before clinical translation. Additionally, Liu et al. reported a very similar hydrogel by replacing the bottom stiff HHy-Br hydrogel with poly (N-isopropylacrylamide-co-acrylic acid-co-initiator) without changing the other procedures, and then further modified by poly (SPMA) to prepare thermo-responsive gradient and layered hydrogels [72]. The design of these similar works inspired by cartilage structure and components provides a novel way to develop cartilage-like hydrogels in the future.

### 3.4. Other Cartilage-Inspired Hydrogels

**Figure 7:**
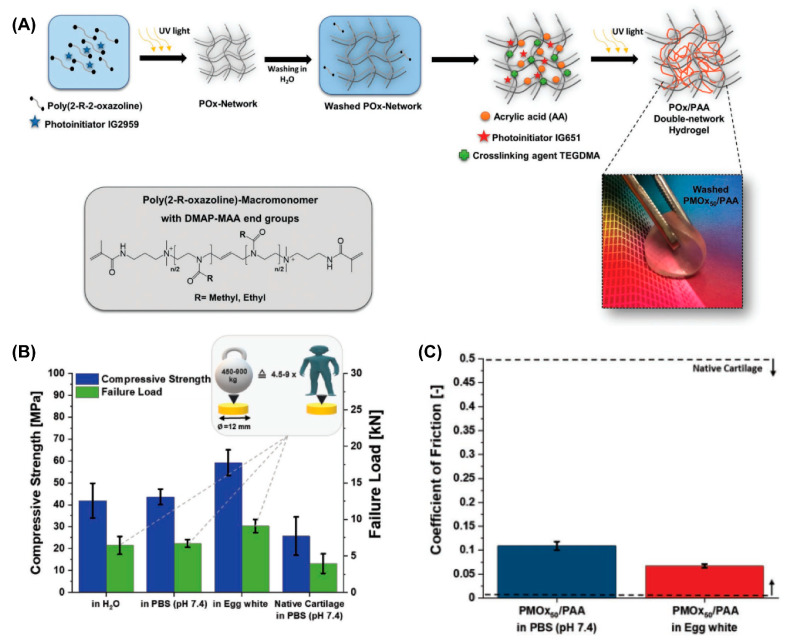
(**A**) Steps in the synthesis of POx/PAA double-network hydrogels. (**B**) Compressive strength and failure load of AC and POx/PAA hydrogels in PBS (pH 7.4). (**C**) The friction coefficient of POx/PAA hydrogels lubricated by PBS (pH 7.4) and egg white. Reprinted with permission from Ref [75]. Copyright 2022, John Wiley and Sons.

Many other hydrogels with cartilage-like features as a substitution for AC have been developed recently. Among them, double-network (DN) hydrogels [60,73] and poly(vinyl alcohol) (PVA)-based hydrogels [74] have shown promising potential due to their high mechanical strength, low friction coefficient, and biocompatibility. P. A. Benitez-Duifn et al. designed an ultrastrong DN hydrogel based on poly(2-oxazoline)s (POx) and non-ionized poly(acrylic acid) (PAA) with stable water-uptake and mechanical performance upon environmental changes [75]. As shown in Figure 7, the first POx network was obtained via the photopolymerization of a monomer of 2-methyl-2-oxazolin or 2-ethyl-2-oxazoline (EtOx), followed by soaking with acrylic acid before the second photopolymerization to obtain the DN hydrogel (POx/PAA hydrogel). As the strong hydrogen bond former, the mechanical performance of PAA-based hydrogels usually significantly decreased in non-acidic conditions due to the deprotonation of carboxylic acid groups in PAA. However, in this POx/PAA hydrogel, the pKa of PAA was shifted to a higher value due to the presence of the POx network, which introduced stable hydrogen bonds between PAA and POx, and thus the mechanical properties were not sacrificed under physiological conditions. When the degree of polymerization of POX was 50, the obtained PMOx50/PAA showed the best stiffness. Given the artificial cartilage, the compressive strength of PMOx50/PAA measured in PBS and egg white were 45 MPa and 60 MPa, respectively, which both exceed that of AC measured in PBS (29.6 ± 8.7 MPa). Additionally, the friction coefficient of PMOx50/PAA sliding against an Al_2_O_3_ ball under the load of 5N (contact pressure: 1.2 MPa) was 0.11 in PBS, and 0.07 when lubricated by egg white.

**Figure 8:**
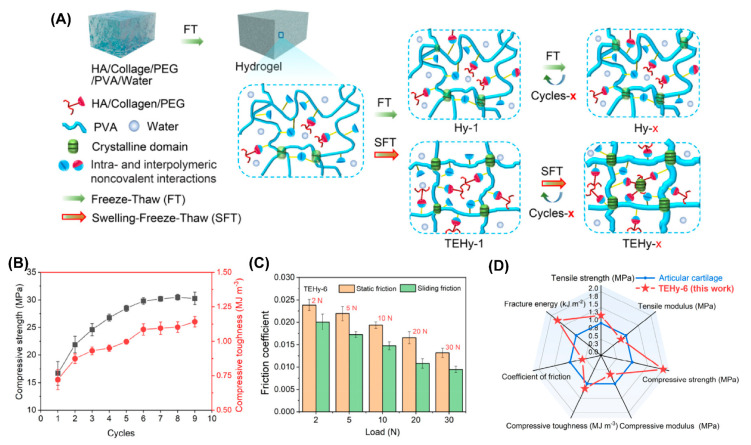
(**A**) Schematic illustration of the fabrication procedures of TEHy-x via the swelling-freeze–thaw method. (**B**) Compressive toughness and compressive strength of TEHy-x under different numbers of FTS cycles. (**C**) Static and sliding friction coefficients of TEHy-6 under different loads. (**D**) Summary and comparison of seven properties of the TEHy-x and AC in a radar chart. Reprinted with permission from Ref [82]. Copyright 2022, American Chemical Society.

PVA has been widely used as a model biomaterial to develop cartilage-like hydrogels due to its excellent biocompatibility, and because it can easily form hydrogels via freeze–thawing repeats [74,76,77]. However, pure PVA hydrogel is not suitable for artificial cartilage due to its poor mechanical performance (especially wear, fragile, and fatigue) under long-standing and multiple cycle loads caused by weakening induced by swelling [78,79]. Recently, Luo et al. developed a PVA/chitosan (CS)/sodium alginate (SA) composited hydrogel (TPCS) that integrated high strength, low friction, and biocompatibility via triple physical crosslinking [80]. TPCS included crystalline regions between the PVA chains, hydrogen bonds between the PVA and CS, and ionic interactions between the CS and SA; thus the obtained TPCS hydrogel exhibited compressive strength as high as 141 MPa. The friction coefficient of TPCS sliding against a steel ball was 0.044, which was the lowest compared with the control groups. They attributed the low friction to the carboxyl groups in the SA, which increased the thickness of the hydration layer between the opposing sliding surfaces. Very recently, they prepared a similar PVA-based hydrogel called HPCS by replacing the SA with sodium hyaluronate (SH), also via the synergy of crystallization, hydrogen bonds, and ionic bonds [81]. One side of HPCS was dipped into the K_2_HPO_4_/CaCl_2_ aqueous solution to mineralize a layer of hybridized hydroxyapatite (HAp), which might promote the combination of hydrogels with the subchondral bone after implantation. The HPCS-HAp hydrogel showed great potential for cartilage replacement due to its high compressive strength (78 MPa), the fact that it is non-swellable in PBS solution, and most importantly, its low friction (friction coefficient as low as 0.024) when sliding against a steel ball. Additionally, Hao et al. reported a swelling-strengthening method of preparing an ultrastrong tough engineered hydrogel (TEHy) based on PVA to meet the mechanical requirements of long-term loads [82], as illustrated in Figure 8. PVA was first dissolved with polyethylene glycol (PEG) in water, and then type I collagen and HA were added, followed by freeze–thaw–swelling (FTS) cycles to obtain TEHy-x (x represents the cycles of FTS). In contrast to the conventional freeze–thaw cycle, the swelling process stretched the PVA chain, and then the stretched network was further crosslinked via freeze–thawing; therefore, the mechanical performance was further improved. TEHy-6 showed high compressive strength (31 MPa) and high load-bearing capacity. It could withstand above 10,000 N load without disrupting the structure. Because of the lubrication layer composed of extruded collagen and HA, the friction coefficient under the load of 30 N was also as low as 0.01 ± 0.002, which was close to that of human cartilage. Notably, after 100,000 cycles of compression, the hydrogel retained its mechanical properties, which suggests the long-term stability of TEHy.

## 4. Conclusions and Perspectives

Hydrogels exhibit significant promise as AC substitutes. The structure and composition of AC have inspired the design and construction of cartilage-like hydrogels with high strength and low friction. Generally, extremely low friction requires a hydrogel with a large degree of hydration, which always decreases the load-bearing capacity. Several strategies inspired by AC have been conceived to fabricate hydrogels to achieve robust load-bearing and extremely low friction simultaneously. In this paper, we summarized the very recent progress in the preparation of such hydrogels that are cartilage-component-inspired and/or cartilage-structure-inspired, as well as others. Although significant progress has been made in the study of cartilage-like hydrogels, there are still some challenges that must be overcome before such hydrogels can be used as cartilage substitutes in clinics. In the future, much more attention should be paid to the following three points: (1) current studies focus on fabricating hydrogels that achieve extremely low friction and high strength, especially high load-bearing capacities; however, the evaluation of these properties should be carried out under realistic simulated conditions, especially considering the fatigue and long-term service in vivo once such hydrogels are applied as AC substitute; (2) the biosafety and degradation, especially the degradation in vivo and its effect on the performance and surrounding tissue, should be taken into consideration; (3) could these cartilage-like hydrogels be bioactive? Introducing specific drugs or growth factors without sacrificing performance could perhaps stimulate the regeneration of AC and thus open up a new avenue for AC regeneration. Summarily, we believe the recent progress in cartilage-like hydrogels presented here, as well as the new hydrogels further inspired by AC, will greatly promote the development of high-strength low-friction hydrogels for AC defect repair.

## Data Availability

Not applicable.
